# A Fast Algorithm for Estimating Two-Dimensional Sample Entropy Based on an Upper Confidence Bound and Monte Carlo Sampling

**DOI:** 10.3390/e26020155

**Published:** 2024-02-10

**Authors:** Zeheng Zhou, Ying Jiang, Weifeng Liu, Ruifan Wu, Zerong Li, Wenchao Guan

**Affiliations:** School of Computer Science and Engineering, Guangdong Province Key Laboratory of Computational Science, Sun Yat-sen University, Guangzhou 510275, China

**Keywords:** sample entropy, Monte Carlo algorithm, upper confidence bound strategy

## Abstract

The two-dimensional sample entropy marks a significant advance in evaluating the regularity and predictability of images in the information domain. Unlike the direct computation of sample entropy, which incurs a time complexity of $O(N^{2})$ for the series with *N* length, the Monte Carlo-based algorithm for computing one-dimensional sample entropy (MCSampEn) markedly reduces computational costs by minimizing the dependence on *N*. This paper extends MCSampEn to two dimensions, referred to as MCSampEn2D. This new approach substantially accelerates the estimation of two-dimensional sample entropy, outperforming the direct method by more than a thousand fold. Despite these advancements, MCSampEn2D encounters challenges with significant errors and slow convergence rates. To counter these issues, we have incorporated an upper confidence bound (UCB) strategy in MCSampEn2D. This strategy involves assigning varied upper confidence bounds in each Monte Carlo experiment iteration to enhance the algorithm’s speed and accuracy. Our evaluation of this enhanced approach, dubbed UCBMCSampEn2D, involved the use of medical and natural image data sets. The experiments demonstrate that UCBMCSampEn2D achieves a 40% reduction in computational time compared to MCSampEn2D. Furthermore, the errors with UCBMCSampEn2D are only 30% of those observed in MCSampEn2D, highlighting its improved accuracy and efficiency.

## 1. Introduction

Information theory serves as a foundational framework for developing tools to represent and manipulate information [1], particularly in the realms of signal and image processing. Within this paradigm, entropy stands out as a key concept, functioning as a metric for quantifying uncertainty or irregularity within a system or dataset [2]. Stemming from Shannon’s pioneering work on entropy [1], subsequent researchers have advanced the field by introducing diverse methods. Notable examples include one-dimensional approximate entropy (ApEn) [3,4], dispersion entropy [5], sample entropy (SampEn) [2], and other innovative approaches. Multiscale entropy, hierarchical entropy, and their variants have been applied to various fields, such as fault identification [6,7] and feature extraction [8], beyond physiological time series analysis.

In 1991, the concept of ApEn was introduced as a method for quantifying the irregularity of time series [3]. ApEn relies on the conditional probability of the negative average natural logarithm, specifically examining the likelihood that two sequences, initially similar at m points, will remain similar at the subsequent point. Addressing the computational challenges associated with self-similar patterns in ApEn, SampEn was subsequently developed, obtaining sampling points using global random sampling to represent the signal, leading to more robust estimations [1]. Notably, in the domain of biomedical signal processing, SampEn has been successfully employed, demonstrating its effectiveness and applicability [9].

Computing SampEn involves enumerating the number of similar templates within a time series, essentially requiring the count of matching template pairs for the given series. The direct computation of SampEn inherently has a computational complexity $O(N^{2})$, where *N* represents the length of the time series under analysis. To expedite this process, kd-tree based algorithms have been proposed for sample entropy computation. These algorithms effectively reduce the time complexity to $O(N^{2-\frac{1}{m+1}})$, where *m* is denoting the template (or pattern) length [10,11]. Additionally, various approaches like box-assisted [12,13], bucket-assisted [14], lightweight [15], and assisted sliding box (SBOX) [16] algorithms have been developed. Nonetheless, the computational complexity for all these algorithms remains at $O(N^{2})$. To tackle the challenge of computational complexity, a rapid algorithm for estimating Sample Entropy using the Monte Carlo algorithm (MCSampEn) has been introduced in [17]. This algorithm features computational costs that are independent of *N*, and its estimations converge to the exact sample entropy as the number of repeated experiments increases. Experimental results reported in [17] demonstrate that MCSampEn achieves a speedup of 100 to 1000 times compared to kd-tree and assisted sliding box algorithms, while still delivering satisfactory approximation accuracy.

However, the MCSampEn algorithm utilizes a random sampling pattern where the importance of each sampling point varies. The application of averaging methods in this context leads to significant fluctuations in errors, resulting in a large standard deviation and slow convergence of the entire process. To address this, we introduce the upper confidence bound (UCB) strategy to set different confidence bounds for various sampling points, assigning varying levels of importance to these points. This approach mitigates errors caused by averaging methods, reduces the standard deviation of the MCSampEn algorithm, accelerates the convergence speed, and significantly improves computational speed.

In this paper, we extend MCSampEn to compute two-dimensional sample entropy, referred to as MCSampEn2D. To mitigate the challenges of convergence brought about by MCSampEn2D, we integrate MCSampEn2D with the upper confidence bound (UCB) strategy to reduce variance [18]. We call this refined algorithm as the UCBMCSampEn2D. By establishing a confidence upper bound for each experimental round, we differentiate the importance of each experiment. The higher the importance of an experiment, the greater its assigned confidence upper bound. Conversely, for experiments with lower confidence upper bounds, our objective is to minimize the errors they introduce to the greatest extent possible.

The UCBMCSampEn2D algorithm is a notable enhancement, enabling swift convergence and minimal errors, even in scenarios with a limited number of sampling points. The UCBMCSampEn2D algorithm eliminates the need for explicit knowledge of the data distribution. By optimistically adjusting the weights for each round of experiments and estimating the upper bound of the expected value, the UCBMASampEn2D algorithm operates without introducing additional computational burden. This algorithm continuously optimizes weights through online learning [19], providing a solution without the requirement for explicit knowledge of the data distribution. In this study, we systematically assess the performance of the UCBMCSampEn2D algorithm across medical image and natural image datasets. Our investigation reveals two primary advantages of the proposed UCBMCSampEn2D algorithm: (1) The UCBMCSampEn2D algorithm places greater emphasis on the importance of different rounds of experiments by assigning importance levels, leading to a reduction in overall errors and faster convergence speed. (2) Leveraging a reinforcement learning approach, the UCBMCSampEn2D algorithm utilizes local optima to represent true entropy. Through the application of upper confidence bounds, it effectively addresses the challenge of determining how to set importance levels.

Further detailed analysis and results will be provided in subsequent sections to expound upon these advantages and demonstrate the effectiveness of the UCBMCSampEn2D algorithm in comparison to conventional methods.

## 2. Fast Algorithms for Estimating Two-Dimensional Sample Entropy

In this section, we introduce the MCSampEn2D and UCBMCSampEn2D algorithms. Before delving into the details of these approaches, we will first establish the key mathematical symbols and fundamental concepts integral to understanding MCSampEn2D. This groundwork provides a clear and comprehensive exposition of both the MCSampEn2D and UCBMCSampEn2D algorithms.

### 2.1. Groundwork of Two-Dimensional Sample Entropy

Let $U={\left\{u_{i,j}\in R\right\}}_{i=1,2,\ldots ,h}^{j=1,2,\ldots ,w}$ be an image of size h×w. For all $k\in \{1,2,\cdots ,h-\left(m_{h}-1)\right\}$ and $l\in \{1,2,\cdots ,w-\left(m_{w}-1\right)\}$, define two-dimensional matrices $X_{k,l}^{m}$ with size $m_{h}\times m_{w}$, named template matrices, by

(1) $${X^{m}}_{k,l}=\left[\begin{array}{cccc} u_{k,l} & u_{k,l+1} & \ldots & u_{k,l+(m_{w}-1)}\\ u_{k+1,l} & u_{k+1,l+1} & \ldots & u_{k+1,l+(m_{w}-1)}\\ \vdots & \vdots & \vdots & \vdots \\ u_{k+(m_{h}-1),l} & u_{k+(m_{h}-1),l+1} & \ldots & u_{k+\left(m_{h}-1\right),l+\left(m_{w}-1\right)} \end{array}\right],$$

where $m=[m_{h},m_{w}]$ is the embedding dimension vector [20]. We also define $X^{m}:=\left\{X_{a,b}^{m}:a\in \left\{1,2,\cdots ,h-\left(m_{h}-1\right)\right\}\;\mathrm{and}\;b\in \left\{1,2,\cdots ,w-\left(m_{w}-1\right)\right\}\right\}$. For all $k,a\in \{1,2,\cdots ,h-\left(m_{h}-1)\right\}$ and $l,b\in \{1,2,\cdots ,w-\left(m_{w}-1\right)\}$, let $d(X_{k,l}^{m},X_{a,b}^{m})$ be the greatest element of the absolute differences between $X_{k,l}^{m}$ and $X_{a,b}^{m}$. We denote by #E the cardinality of a set *E*. Then, for fixed *k* and *l*, we count $\#\{X_{a,b}^{m}\in X^{m}:d\left(X_{k,l}^{m},X_{a,b}^{m}\right)\leq r\;\mathrm{and}\;{\left(k-a\right)}^{2}+{\left(l-b\right)}^{2}\neq 0\}$, and compute

(2) $$\phi_{k,l}^{m}\left(r\right)=\frac{\#\{X_{a,b}^{m}\in X^{m}:d\left(X_{k,l}^{m},X_{a,b}^{m}\right)\leq r\;\mathrm{and}\;{\left(k-a\right)}^{2}+{\left(l-b\right)}^{2}\neq 0\}}{\left(h-m_{h}\right)\left(w-m_{w}\right)-1},$$

where *r* is the predefined threshold (tolerance factor). We also define $\phi^{m}\left(r\right)$ as

(3) $$\phi^{m}\left(r\right)=\frac{1}{\left(h-m_{h}\right)\left(w-m_{w}\right)}\sum_{k=1}^{k=h-m_{h}}\sum_{l=1}^{l=w-m_{w}}\phi_{k,l}^{m}\left(r\right).$$

Finally, SampEn2D is defined as follows [20]:

(4) $$\mathrm{SampEn}2D\left(U,m,r\right)=-\log\frac{\phi^{m+1}\left(r\right)}{\phi^{m}\left(r\right)},$$

where $m+1=[m_{h}+1,m_{w}+1]$. The parameter m indicates the size of the matrices, which are analyzed or compared along images. In this study, [20,21], m is chosen to obtain squared template matrices, and let $m_{h}=m_{w}\in Z$. For all w,h∈Z, we denote $Z_{w,h}:=\left\{\left(i,j\right)\in Z^{2}:1\leq i\leq w,1\leq j\leq h\right\}$. The process for computing SampEn2D(U,m,r) is summarized in Algorithm 1.

The parameter *r* is selected to strike a balance between the quality of the logarithmic likelihood estimates and the potential loss of signals or image information. If *r* is chosen to be too small (less than 0.1 of the standard deviation of an image), it leads to poor conditional probability estimates. Additionally, to mitigate the influence of noise on the data, it is advisable to opt for a larger *r*. Conversely, when *r* exceeds 0.4 of the standard deviation, excessive loss of detailed data information occurs. Therefore, a trade-off between large and small *r* values is essential. For a more in-depth discussion on the impact of these parameters in SampEn2D, please refer to [20].
**Algorithm** **1** Two-dimensional sample entropy**Require:** Sequence $U:=\{u_{i,j}:1\leq i\leq w,1\leq j\leq h\}$, $s\subset Z_{w,h}$ template length *m* and  threshold *r*.1:**procedure** SampEn2D(U,s,m,r)2:    Set count=0,3:    Set $N_{0}=\#s$,4:    **for** i=1 to $N_{0}$ **do**5:        **for** j=i to $N_{0}$ **do**6:           $\left(k,l\right)=s_{i}$; $\left(a,b\right)=s_{j}$,7:           $X_{i,j}^{m}=U\left[i:i+m-1\right]\left[j:j+m-1\right]$,8:           $X_{a,b}^{m}=U\left[a:a+m-1\right]\left[b:b+m-1\right]$,9:           **if** $d(X_{i,j}^{m},X_{a,b}^{m})\leq r$ **then**10:               count=count+1,11:    **return** count

### 2.2. A Monte Carlo-Based Algorithm for Estimating Two-Dimensional Sample Entropy

The SampEn is fundamentally defined as −log(B/A), where *B* represents the number of matching template pairs of length *m*, and *A* represents the number of matching template pairs of length m+1. The most computationally intensive step in calculating SampEn involves determining the ratio B/A for templates of lengths *m* and m+1. Notably, $\frac{A}{N(N-1)}$ (resp. $\frac{B}{N(N-1)}$) denotes the probability of template matches of length *m* (resp. m+1), and the ratio B/A can be interpreted as a conditional probability. The statement indicates that the computation time of the MCSampEn method becomes independent of the data size and, instead, depends on the number of sampling points $N_{0}$ and the number of repetitions $N_{1}$. This complexity is denoted as $O(N_{1}\left(N_{0}+N_{0}^{2}\right))$ [17].

The objective of the MCSampEn algorithm [17] is to approximate this conditional probability for the original dataset by considering the conditional probability of a randomly subsampled dataset. Specifically, the MCSampEn randomly selects $N_{0}$ templates of length *m* and $N_{0}$ templates of length m+1 from the original time series. It subsequently computes the number of matching pairs in the selected templates of length *m* (resp. m+1), denoted as $\tilde{A}$ (resp. $\tilde{B}$). This selection process is repeated $N_{1}$ times, and the average value of $\left\{{\tilde{A}}_{k}:k=1,2,\ldots ,N_{1}\right\}$ (resp. $\left\{{\tilde{B}}_{k}:k=1,2,\ldots ,N_{1}\right\}$), represented as ${\bar{A}}_{N_{1}}$ (resp. ${\bar{B}}_{N_{1}}$), is then calculated. Finally, $-\log({\bar{B}}_{N_{1}}/{\bar{A}}_{N_{1}})$ is employed to approximate the complexity measurement −log(B/A) for the time series. The entire process can be expressed succinctly using the following formula:

$${\bar{A}}_{N_{1}}:=\frac{1}{N_{1}}\sum_{k=1}^{N_{1}}{\tilde{A}}_{k},\;\mathrm{and}\;{\bar{B}}_{N_{1}}:=\frac{1}{N_{1}}\sum_{k=1}^{N_{1}}{\tilde{B}}_{k},$$

(5) $$\begin{array}{c} \mathrm{MCSampEn}:=-\log\frac{{\bar{B}}_{N_{1}}}{{\bar{A}}_{N_{1}}}, \end{array}$$

where ${\tilde{A}}_{k}$ (resp. ${\tilde{B}}_{k}$) means the number of matching pairs in the selected templates of length *m* (resp. m+1) in in the *k*-th experiment.

When extending the MCSampEn algorithm to process two-dimensional data, a random sampling step is essential at the outset to acquire *N* data points. The technique employs a specific sampling strategy to sample $N_{0}$ positive integer sets, denoted as $V=\{v_{i}:i=1,2,\cdots ,N_{0}\}$ and $v_{i}\leq \left(h-m_{h}\right)\times \left(w-m_{w}\right)$. Subsequently, for each $v_{i}$, compute $k=v_{i}/h$ and $l=v_{i}\%h$, which indicates a two-dimensional matrix $X_{k,l}^{m}\in X^{m}$. Then, we randomly select $N_{0}$ templates of size m and $N_{0}$ templates of length m+1 from the original two-dimensional data. It subsequently computes the number of matching pairs, with tolerant factor *r*, in the selected templates of length m (resp. m+1), denoted as ${\tilde{\phi }}^{m}$ (resp. ${\tilde{\phi }}^{m+1}$). This selection process is repeated $N_{1}$ times, and the average value, represented as ${\bar{\phi }}_{N_{1}}^{m}$ (resp. ${\bar{\phi }}_{N_{1}}^{m+1}$), is then calculated. Replacing ${\bar{A}}_{N_{1}}$ and ${\bar{B}}_{N_{1}}$ by ${\bar{\phi }}_{N_{1}}^{m}$ and ${\bar{\phi }}_{N_{1}}^{m+1}$ in (5), respectively, we obtain an approximation of SampEn2D. This process is summarized in Algorithm 2, which calls Algorithm 1.
**Algorithm** **2** Two-dimensional Monte Carlo sample entropy (MCSampEn2D)**Require:** Sequence $U:=\{u_{i,j}:1\leq i\leq w,1\leq j\leq h\}$, template length *m*, threshold *r*,  Sample numbers $N_{0}$ and experimental rounds $N_{1}$.1:**procedure** MCSampEn2D($U,m,r,N_{0},N_{1}$)2:    Set ${\bar{\phi }}_{N_{1}}^{m}=0$ and ${\bar{\phi }}_{N_{1}}^{m+1}=0$,3:    **for** k=1 to $N_{1}$ **do**4:        Set $\mathrm{Cor}=\{\left(h_{s},w_{s}\right):1\leq s\leq N_{0}\}$ where $h_{s}$ and $w_{s}$ are selected on U pixel coordinates with uniform distribution,5:        Compute ${\tilde{\phi }}_{k}^{m}$ by calling SampEn2D(U,Cor,m,r),6:        Compute ${\tilde{\phi }}_{k}^{m+1}$ by calling SampEn2D(U,Cor,m+1,r),7:        ${\bar{\phi }}_{N_{1}}^{m}={\bar{\phi }}_{N_{1}}^{m}+\frac{1}{N_{1}}\sum_{k=1}^{N_{1}}{\tilde{\phi }}_{k}^{m}$,8:        ${\bar{\phi }}_{N_{1}}^{m+1}={\bar{\phi }}_{N_{1}}^{m+1}+\frac{1}{N_{1}}\sum_{k=1}^{N_{1}}{\tilde{\phi }}_{k}^{m+1}$9:    $entropy=-\log\frac{{\bar{\phi }}_{N_{1}}^{m+1}}{{\bar{\phi }}_{N_{1}}^{m}}$,10:    **return** entropy

It is easy to check the computational cost of MCSampEn2D is $O(N_{1}N_{0}^{2})$ when m is fixed. Through a proof process similar to that of Theorem 5 in [17], we can see that the output of MCSampEn2D, $-\log\frac{{\bar{\phi }}_{N_{1}}^{m+1}}{{\bar{\phi }}_{N_{1}}^{m}}$, is approximating the output of SampEn2D with the rate $O(N_{1}^{-1}\log N_{1})$ in the sense of almost sure convergence when $N_{0}$ is fixed. Furthermore, in our examination of MCSampEn2D, we observed variability in the significance of the randomly subsampled dataset. This is manifested as large fluctuations in the errors between the values of ${\tilde{\phi }}^{m}$ (or ${\tilde{\phi }}^{m+1}$) obtained in each of the $N_{1}$ rounds of experiments and their respective average values, ${\bar{\phi }}_{N_{1}}^{m}$ (or ${\bar{\phi }}_{N_{1}}^{m+1}$). Such fluctuations amplify the errors in the output of MCSampEn2D. This phenomenon made us realize that if we could capture the importance of different experimental rounds and use this importance to calculate a weighted average of ${\tilde{\phi }}^{m}$ (or ${\tilde{\phi }}^{m+1}$) obtained in the $N_{1}$ rounds of experiments, we could achieve a faster algorithm than MCSampEn2D.

### 2.3. Monte Carlo Sample Entropy Based on the UCB Strategy

The UCB strategy is a refinement in the field of optimization, particularly tailored for the intricate problem of the multi-armed bandit. This problem, often conceptualized as a machine or ’gambler’ with multiple levers (or ’arms’), each offering random rewards on each interaction, demands finding the optimal lever that maximizes reward yield with minimal experimentation. The UCB strategy’s core principle is to meticulously balance the pursuit of exploration and exploitation. Exploration, in this context, signifies the willingness to experiment with untested options, while exploitation underscores the preference to capitalize on the rewards offered by gamblers with a proven track record of high performance. This balancing act, inherent in the UCB strategy, aids in optimizing the overall reward yield by efficiently determining the optimal balance between exploring new options and exploiting known high-reward choices, thereby minimizing the number of trials required to reach the optimal solution [22,23].

In the preceding section, we presented the MCSampEn2D for estimating two-dimensional sample entropy. The precision of MCSampEn2D is contingent upon factors such as the number of experimental rounds, denoted as $N_{1}$, the quantity of sampled points, $N_{0}$, and the representativeness of these sampled points. Given that distinct experimental rounds involve the selection of different sampled points, their representativeness inherently varies. In Equation (5), the weight assigned to each round is statically set to $1/N_{1}$. To enhance accuracy, we can dynamically adjust the weights based on the representativeness of the sampled points in each round.

The adjustment of weights based on the representativeness of the sampled points serves to refine the estimation of sample entropy in each round, thereby enhancing the overall precision of the algorithm. This approach aims to ensure that the sampling process more accurately reflects the inherent characteristics of the dataset.

In the realm of decision-making under limited resources, the multi-armed bandit problem represents a classic challenge in reinforcement learning, involving a series of choices with the goal of maximizing cumulative rewards. The UCB strategy emerges as a crucial approach for tackling the multi-armed bandit problem. Its central concept revolves around dynamically assessing the potential value of each choice [24,25], seeking a balance between the exploration of unknown options and the exploitation of known ones.

At the core of the UCB strategy is the principle of making selections based on the upper confidence bounds. In this strategy, each arm represents a potential choice, and the true reward value of each arm remains unknown. In Algorithm 2, we refer to the process executed from step 5 to step 6 as one epoch. Under the UCB strategy, we conceptualize each epoch as an arm, which means that $N_{1}$ epochs represent $N_{1}$ arms, and dynamically update its upper confidence bound based on a designated reward function for each epoch. The UCB strategy involves the following three steps at each time step.

Firstly, the average reward for each round of epochs is calculated by

$$\hat{q}\left(i\right)=\frac{X(i)}{K(i)},$$

where $\hat{q}\left(i\right)$ signifies the average reward for the *i*-th round. In our design, each epoch is utilized only once per round, thus we set K(i)=1 for all $i\in \{1,2,\ldots ,N_{1}\}$. Here, X(i) represents the reward for the current epoch, estimated from historical data.

To design X(i), we define the following notations. For all $1\leq i\leq N_{1}$, let ${\bar{r}}_{i}$ be the average of the sample entropy of the preceding i−1 rounds, $r_{i}$, be the entropy computed for the ongoing round, and $e_{i}:={\bar{r}}_{i-1}-r_{i}$, where ${\bar{r}}_{0}:=0$. Considering that different images inherently possess distinct entropies, we introduce the error ratio $er_{i}=\frac{e_{i}}{{\bar{r}}_{i-1}}$ to characterize the error situation for the *i*-th round. This ratio is then used as an input to the reward function X(i). In formulating the reward function, our objective is to assign higher rewards to rounds where errors are closer to 0. Multiple choices exist for defining the reward function *R*, provided that it aligns with the design specifications of the particular scenario. Various mathematical functions, such as the cosine function and normal distribution function, among others, are viable options for constructing the reward function. The selection of a specific function is contingent upon its ability to meet the desired criteria and effectively capture the intended behavior in the context of the given problem. Then, we set the average reward $\hat{q}\left(i\right)$ for the *i*-th round of epochs formula as

(6) $$\hat{q}\left(i\right)=X\left(i\right):=a\times R\left(b\times er_{i}\right),$$

where *a* is a scaling factor for the reward and *b* controls the scale of $er_{i}$.

Secondly, we calculate the upper confidence limit boundary for each round of epochs by

(7) $${\mathrm{ucb}}_{i}=\hat{q}\left(i\right)+c\sqrt{2\ln(i)},$$

where ${\mathrm{ucb}}_{i}$ represents the upper confidence bound for the *i*-th round and set K(i) as a constant equal to 1, we use a parameter *c* to control the degree of exploration. Denote $\tilde{U}:=\left\{{\mathrm{ucb}}_{i}:i\in \left\{1,2,\ldots ,N_{1}\right\}\right\}$, which is the set of UCB.

Thirdly, for the set $\tilde{U}$, we employ the softmax function to determine the proportional weight each epoch round should have, thereby replacing the average proportion used in MCSampEn2D. We then calculate ${\bar{A}}_{\mathrm{Ucb}}$ and ${\bar{B}}_{\mathrm{Ucb}}$ for the UCBMCSampEn2D by

$${\bar{A}}_{\tilde{U}}=\sum_{k=1}^{N_{1}}S_{k}{\tilde{A}}_{k},\;{\bar{B}}_{\tilde{U}}=\sum_{k=1}^{N_{1}}S_{k}{\tilde{B}}_{k},$$

where $S=\mathrm{softmax}(\tilde{U})$ and the UCBMCSampEn can be calculated by

$$\mathrm{UCBMCSampEn}=-\log\frac{{\bar{B}}_{\tilde{U}}}{{\bar{A}}_{\tilde{U}}}.$$

The pseudocode for the entire UCBMCSampEn is outlined in Algorithm 3.
**Algorithm** **3** Monte Carlo sample entropy based on UCB strategy**Require:** Sequence $U:=\{u_{i,j}:1\leq i\leq w,1\leq j\leq h\}$, template length *m*, threshold *r*,  Sample numbers $N_{0}$ and epoch numbers $N_{1}$.1:**procedure** UCBMCSampEn($U,m,r,N_{0},N_{1}$)2:    Set ${\bar{A}}_{\tilde{U}}=0$ and ${\bar{B}}_{\tilde{U}}=0$,3:    **for** k=1 to $N_{1}$ **do**4:        Set $\mathrm{Cor}=\{\left(h_{s},w_{s}\right):1\leq s\leq N_{0}\}$ where $h_{s}$ and $w_{s}$ are selected on U pixel coordinates with uniform distribution,5:        Compute ${\tilde{A}}_{k}$ by calling SampEn2D(U,Cor,m,r),6:        Compute ${\tilde{B}}_{k}$ by calling SampEn2D(U,Cor,m+1,r),7:        Compute $e_{k}=-\log\frac{{\tilde{B}}_{k}}{{\tilde{A}}_{k}}$, $e_{m}=\frac{1}{k}\sum_{1}^{k}e_{k}$,8:        Compute $er_{k}=\frac{e_{m}-e_{k}}{e_{m}}$,9:        Compute ${\mathrm{ucb}}_{k}=a\times R\left(b\times er_{k}\right)+c\sqrt{2\ln(i)}$,10:    Set $\tilde{U}=\left\{{\mathrm{ucb}}_{k}:k\in \left(1,2,\cdots ,N_{0}\right)\right\}$,11:    Set $S=\mathrm{softmax}(\tilde{U})$,12:    **for** k=1 to $N_{1}$ **do**13:        ${\bar{A}}_{\tilde{U}}={\bar{A}}_{\tilde{U}}+{\tilde{A}}_{k}\times S_{k}$,14:        ${\bar{B}}_{\tilde{U}}={\bar{B}}_{\tilde{U}}+{\tilde{B}}_{k}\times S_{k}$,    $entropy=-\log\frac{{\bar{B}}_{\tilde{U}}}{{\bar{A}}_{\tilde{U}}}$15:    **return** entropy

Because the averaging strategy in the MCSampEn method does not consider the varying importance of sampling points across different epochs, it can result in significant errors. Although the UCB strategy introduces bias [26], it can mitigate the errors introduced by the averaging strategy, transforming the uniform strategy in the original method into an importance-based strategy. This adjustment aligns the sampling more closely with the actual characteristics of the data.

## 3. Experiments

This section is dedicated to thoroughly evaluating the effectiveness of the UCBMCSampEn algorithm by implementing it across various domains. All of our experiments were carried out on the Linux platform. The platform utilizes an Intel(R) Xeon(R) Gold 6248R processor with a clock frequency of 3.00 GHz.

### 3.1. Datasets

**Figure 1:**
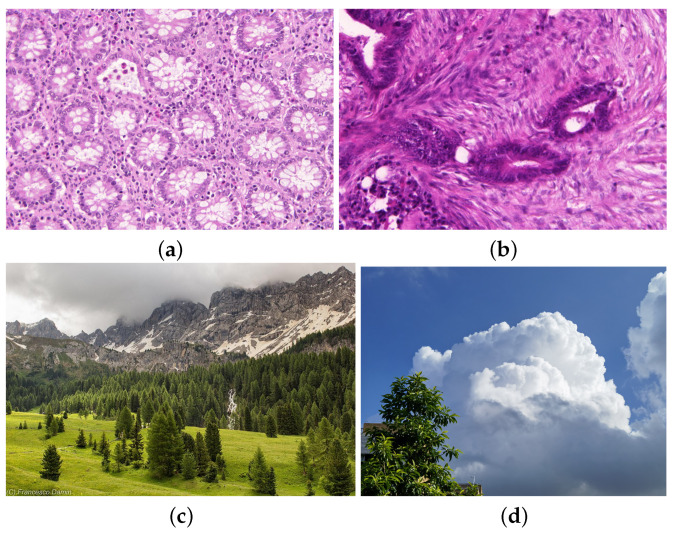
Examples of reference images include: images (**a**,**b**) in the Warwick QU Dataset, which represent benign and malignant cases, respectively, each with a size of 580×440; image (**c**) is a natural image with a size of 775×522; and image (**d**), called the wallpaper, with a size of 3000×3000, is used to verify the method’s performance on large-scale data.

To facilitate a thorough investigation, our experiments incorporate a range of sequences characterized by distinct features. These sequences are categorized primarily into datasets encompassing medical image data and natural image data. The medical image dataset, named Warwick QU dataset, is derived from the Colon Histology Images Challenge Contest for Gland Segmentation (GlaS), organized by MICCAI 2015, where participants developed algorithms for segmenting benign and diseased tissues [27]. It contains 165 samples extracted from H&E-stained colon histology slides. The slides were derived from 16 different patients, from which malignant and benign visual fields were extracted. The dataset example is illustrated in Figure 1.

### 3.2. Main Results

**Figure 2:**
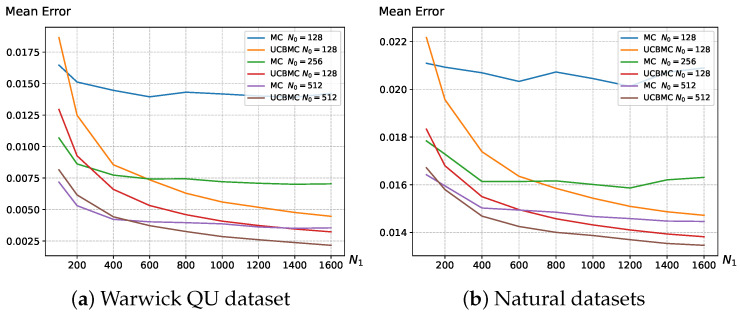
(**a**) depicts the average error variation in MCSampEn2D and UCBMCSampEn2D experiments on the Warwick QU dataset with changing $N_{1}$, where parameters are set to m=2 and r=0.3; (**b**) depicts the average error variation in MCSampEn2D and UCBMCSampEn2D experiments on natural datasets with changing $N_{1}$, where parameters are set to m=2, r=0.3, a=5 and b=1. The reward function is set as the cosine function.

In this section, we validate the effectiveness of the UCBMCSampEn2D, comparing its computational time and computational error with the MCSampEn2D algorithm. Figure 2 illustrates the variation in entropy mean error with the number of epochs $N_{1}$ using sampling points $N_{0}$ on the Warwick QU dataset and natural image dataset. The formula for calculating the mean error is as follows:

$$\mathrm{MeanError}=\frac{\sum_{i=1}^{N}\left|d_{i}-e_{i}\right|}{W},$$

where *W* represents the number of images in the dataset, $d_{i}$ is the entropy of the *i*-th image calculated using the direct algorithm, and $e_{i}$ is the entropy of the *i*-th image calculated using the MCSampEn2D (or UCBMCSampEn2D) algorithm. The results demonstrate that the UCBMCSampEn2D algorithm converges more quickly, significantly reducing the error in comparison to the MCSampEn2D algorithm.

**Figure 3:**
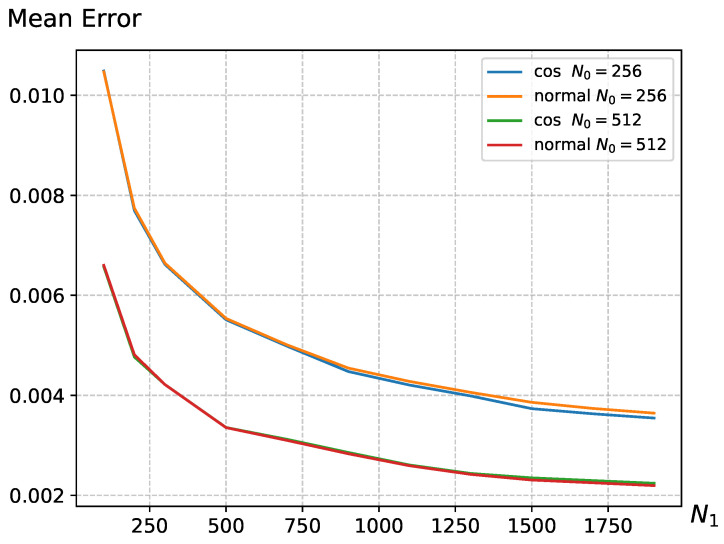
The average error variation in different reward function *R* with changing $N_{1}$ on the Warwick QU dataset, where parameters are set to m=2 and r=0.3. The parameters for the cosine function in the reward function are set as a=8 and b=0.5, while for the normal distribution function, the parameters are set as a=8 and b=2.

In Equation (6), we discussed that the reward function *R* offers flexibility, providing multiple choices to meet the design requirements of the scenario. In Figure 3, we conducted experiments using two different reward functions, and the results indicate that, with reasonable parameter settings, different reward functions *R* exhibit similar trends in average error changes. This suggests that the UCB strategy has a certain degree of generality and is not confined to specific forms. The remaining experiments were all conducted using the cosine function.

Figure 2 provides a detailed view of the situation with sampling points set at $N_{0}=128$. Based on the experimental results, it is evident that the UCBMCSampEn2D algorithm demonstrates more rapid convergence with an increase in the number of experiments ($N_{1}$) compared to the MCSampEn2D. In Figure 2, with $N_{0}=128$, the UCBMCSampEn2D algorithm initially exhibits a larger error during the first 150 rounds of experiments. However, as the number of experiments increases, the UCBMCSampEn2D algorithm quickly reduces the error and achieves a lower convergence error than the MCSampEn2D. This substantial improvement in accuracy is consistently observed across various values of $N_{0}$.

This phenomenon is elucidated in Section 2.3 of our algorithm, where the first *i* rounds of epochs are utilized to calculate the average entropy, simulating the true entropy. When *i* is small, there is not enough historical data to support it, the average entropy at this point introduces a relatively large error. However, since the entropy calculated from the previous *i* rounds is relatively close, the reward obtained for these initial rounds in (6) tends to be too high, leading to a larger error. As *i* increases, the average entropy more closely approximates the true entropy, and the weights assigned subsequently become more accurate in reflecting the real situation, thereby enabling the algorithm to converge more effectively.

Table 1 details the specifics of the UCBMCSampEn2D algorithm. Throughout the experiment, we maintained a consistent template length of m=2 and a fixed similarity threshold of r=0.3. Adjustments were made only to the number of sampling points, $N_{0}$, and the number of epochs, $N_{1}$.

In Table 2, under identical time constraints and with the same values of $N_{0}$ and $N_{1}$, the UCBMCSampEn2D algorithm demonstrates an error that is only 30% of the error observed with the MCSampEn2D algorithm when $N_{0}$ is small. Additionally, when $N_{0}$ is large, the UCBMCSampEn algorithm consistently outperforms the MCSampEn2D algorithm in terms of error reduction.

We can see that the MCSampEn2D algorithm has a significantly improved computation speed compared to the traditional SampEn2D, with an acceleration ratio exceeding a thousand-fold. Additionally, we conducted a time comparison between the UCBMCSampEn2D algorithm and the MCSampEn2D algorithm, setting the error below $5\times 10^{-3}$. The UCBMCSampEn2D algorithm also demonstrated advantages, as shown in Table 2. In comparison to the MCSampEn2D algorithm, the UCBMCSampEn2D algorithm reduced the computation time by nearly 40%. Moreover, for larger-sized sequences, the UCBMCSampEn2D algorithm exhibited a significant advantage over the MCSampEn2D algorithm in terms of computation time and error.

Simultaneously, we conducted numerical experiments on randomly generated binary images with a size of 512×512. The generation function MIX(p)[20] had a parameter p=0.9. The results are shown in Table 3. Our method continues to demonstrate advantages even in the presence of high data randomness.

## 4. Discussion

### 4.1. Analysis of the UCB Strategy

In Section 2.2, we observed that the MCSampEn2D algorithm utilizes a random sampling method to select $N_{0}$ points and computes sample entropy across $N_{1}$ epoch numbers by averaging the outcomes. The chosen points’ proximity to key image features affects their representativeness for the entire image, thereby influencing their relative importance. When sampled points accurately capture critical information, the error in that epoch number is reduced, resulting in sample entropy values that more closely approximate the true entropy. On the other hand, if the points fail to effectively capture information in the image, the resultant error in that round is magnified. The MCSampEn2D algorithm, which simply averages results across all rounds without weighing their importance, is adversely affected by these variations. This situation results in larger errors during convergence, particularly influenced by the number of sampled points $N_{0}$. Additionally, due to its inherent random sampling method, the standard deviation of the MCSampEn2D algorithm’s results varies significantly with each epoch, leading to slower convergence and extended computation times.

**Figure 4:**
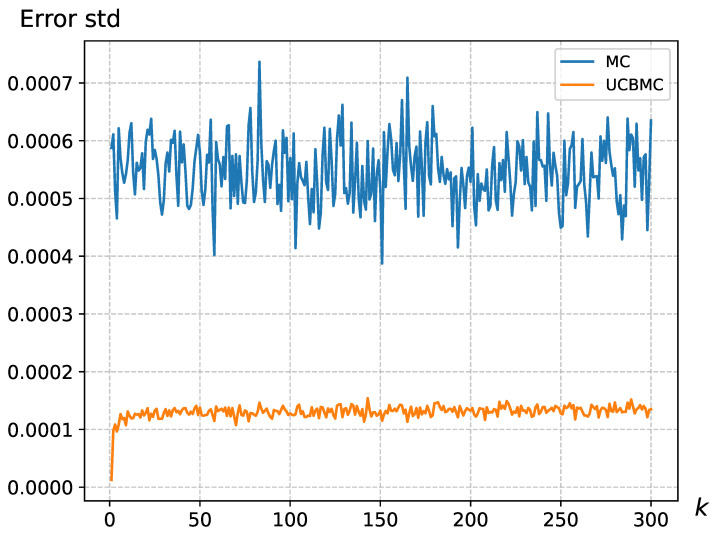
The error standard deviation variation for the wallpaper, where $N_{0}=128$, $N_{1}=300$, where the UCB parameters were set at a=8 and b=1.

**Figure 5:**
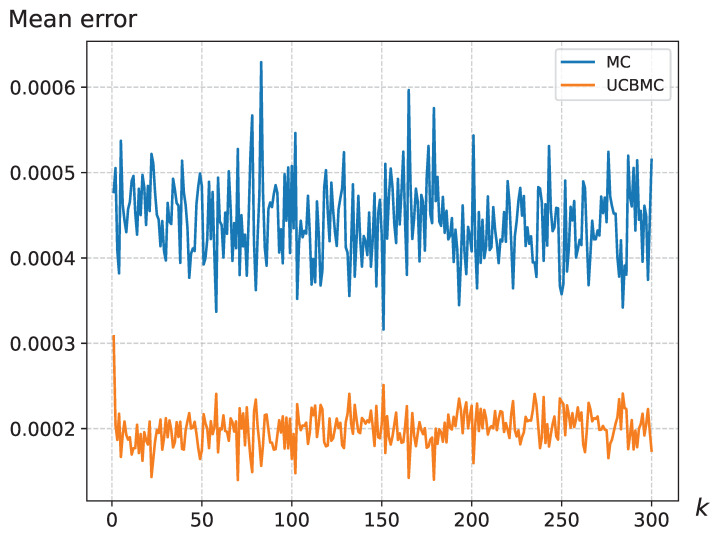
The mean error variation for the wallpaper, where the UCB parameters were set at a=8 and b=1.

We have addressed the MCSampEn2D algorithm’s limitation in accurately reflecting the importance of epochs by integrating the UCB strategy. This strategy assigns significance to different epochs, thus modulating their individual impact on the final result. To compare the effectiveness of these approaches, we conducted 50 epochs each for MCSampEn2D and UCBMCSampEn2D using images from a natural dataset, specifically of size 3000×3000. We calculated the average and standard deviation of the error for the *k*-th round ($k\in \{1,2,\cdots ,N_{1}\}$). The results, displayed in Figure 4 and Figure 5, reveal that the standard deviation of MCSampEn2D shows significant fluctuations across different rounds, while UCBMCSampEn2D maintains a more consistent performance. Furthermore, the error values for MCSampEn2D are consistently higher compared to those of UCBMCSampEn2D. This demonstrates that UCBMCSampEn2D not only achieves smaller errors than MCSampEn2D within the same timeframe but also effectively mitigates the issue of MCSampEn2D’s inability to adequately capture the importance of each epoch.

Furthermore, we observed larger errors in the initial rounds of epochs with UCBMCSampEn2D. This can be attributed to the fact that the average entropy, serving as a temporary anchor, does not initially consider the historical context. Consequently, this leads to an overly high reward, $\hat{q}\left(i\right)$, in Equation (6) which, in turn, causes the confidence bound, ${\mathrm{ucb}}_{i}$, in Equation (7) to be inconsistent. As a result, this inconsistency contributes to larger errors in the early rounds of epochs.

### 4.2. The Impact of Parameters on the UCB Strategy

**Figure 6:**
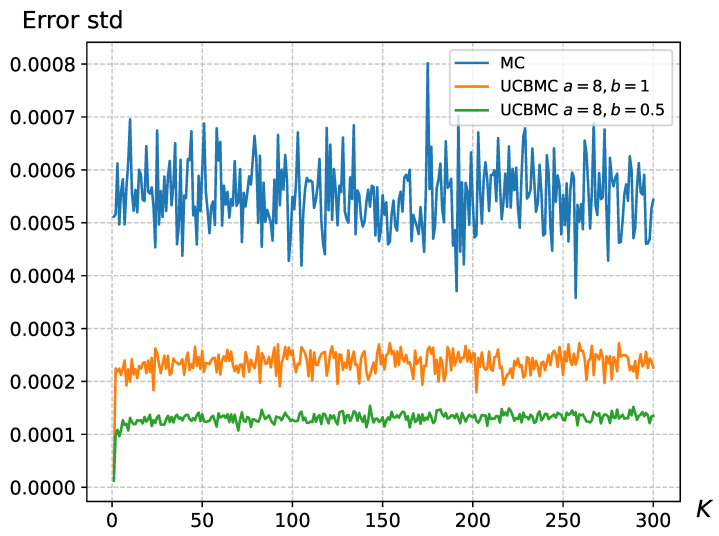
The error standard deviation variation for the wallpaper, where $N_{0}=128$ and $N_{1}=300$.

In Section 2.3, where we introduced the formula for UCBMCSampEn2D, it was observed that the parameters *a* and *b* significantly impact the convergence speed and error of the algorithm. Since $er_{i}$ in (6) reflects the proportion of bias, an unreasonable bias proportion could render the reward function ineffective. We conducted 50 experiments using wallpaper images and computed the standard deviation of the error for the *k*-th round ($k\in 1,2,\cdots ,N_{1}$), as shown in Figure 6. The results in Figure 6 demonstrate that the effectiveness of UCBMCSampEn2D is influenced by the parameters *a* and *b*. Appropriate selection of the values for *a* and *b* can reduce the standard deviation of the errors. Based on our experimental tests, we recommend using a∈(7,9) and b∈(0.4,0.6). However, adjustments may still be necessary based on the image size and the number of sampling points $N_{0}$.

### 4.3. The Application of Sample Entropy in Medical Image Dataset

In the Warwick QU dataset, all pathological slices can be categorized into two classes: Benign and Malignant. We computed the entropy using the UCBMCSampEn2D algorithm for the dataset and utilized an SVM for training and classification, yielding the results presented in Table 4. It is evident that the entropy calculated by the UCBMCSampEn2D algorithm exhibits distinct trends for the two types of pathological slices, demonstrating potential in pathological slice diagnosis and positioning it as a viable feature for aiding future work in this field. The findings suggest that sample entropy can serve as a valuable supplementary characteristic in the context of pathological diagnosis.

## 5. Conclusions

This paper introduces two accelerated algorithms for estimating two dimensional sample entropies, termed MCSampEn2D and UCBMCSampEn2D. These algorithms were rigorously tested on both medical and natural datasets. The study’s significance is manifold: firstly, the MCSampEn2D algorithm, an extension of the MCSampEn algorithm, substantially improves the computational efficiency for two-dimensional sample entropy. Further, we delve into the convergence challenges faced by the MCSampEn2D algorithm and adopt the UCB strategy to mitigate these issues. This strategy, as applied in our study, prioritizes the varying significance of different epochs, with its upper confidence bounds effectively mirroring this importance. The experiments detailed in Section 3 validate the efficacy of both the MCSampEn2D and UCBMCSampEn2D algorithms.

Overall, due to the UCBMCSampEn2D algorithm’s impressive performance in computing sample entropy, it demonstrates considerable promise for analyzing diverse images while minimizing computational time and error.

## Figures and Tables

**Table 1:** The mean error comparison among different algorithms under the same amount of time. The UCB parameters were set at a=8 and b=1.

Method	$N_{0}$/$N_{1}$	Mean Error (Proportion)	Mean Time (s)
SampEn2D	/	/	402.98
	128/1300	$13.21\times 10^{-3}\;\left(0.906\times 10^{-3}\right)$	4.91
MCSampEn2D	256/1100	$6.78\times 10^{-3}\;\left(0.465\times 10^{-3}\right)$	4.29
	512/900	$3.69\times 10^{-3}\;\left(0.253\times 10^{-3}\right)$	4.87
	128/1300	$4.80\times 10^{-3}\;\left(0.329\times 10^{-3}\right)$	4.91
UCBMCSampEn2D	256/1100	$3.96\times 10^{-3}\;\left(0.271\times 10^{-3}\right)$	4.29
	512/900	$2.78\times 10^{-3}\;\left(0.191\times 10^{-3}\right)$	4.87

**Table 2:** The comparison of time and error for image (d) in Figure 1 under different methods.

	SampEn2D	MCSampEn2D	UCBMCSampEn2D
time(s)	161,322	1.5816	0.9057
error	/	$1.499\times 10^{-3}$	$0.442\times 10^{-3}$

**Table 3:** The comparison of time and error for randomly generated binary images under different methods.

	SampEn2D	MCSampEn2D	UCBMCSampEn2D
time (s)	230.546	1.4046	0.9773
error	/	$5.016\times 10^{-3}$	$3.362\times 10^{-3}$

**Table 4:** The UCBMCSampEn2D results for some different categories of pathological slices in Warwick QU dataset.

Image Name	UCBMCSampEn2D	Benign or Malignant
testB_1	0.317812	Benign
train_15	0.529283	Benign
train_47	0.672241	Benign
testA_17	0.762295	Benign
testA_24	1.06252	Benign
testA_57	2.37266	Malignant
testA_59	2.1973	Malignant
testA_8	2.36954	Malignant
testA_19	2.10283	Malignant
testB_7	2.07255	Malignant

## Data Availability

The numerical experiments in this work utilize the following datasets: Kaggle Dataset Landscape Pictures is available at https://www.kaggle.com/datasets/arnaud58/landscape-pictures; Warwick QU Dataset is available at https://warwick.ac.uk/fac/cross_fac/tia/data/glascontest/download; Wall paper is available at https://t.bilibili.com/894092636358443014.

## References

[1] Shannon C.E. (2001). A Mathematical Theory of Communication. Assoc. Comput. Mach..

[2] Richman J.S., Moorman J.R. (2000). Physiological time-series analysis using approximate entropy and sample entropy. Am. J. Physiol. Heart Circ. Physiol..

[3] Pincus S.M. (1991). Approximate entropy as a measure of system complexity. Proc. Natl. Acad. Sci. USA.

[4] Tomčala J. (2020). New fast ApEn and SampEn entropy algorithms implementation and their application to supercomputer power consumption. Entropy.

[5] Rostaghi M., Azami H. (2016). Dispersion entropy: A measure for time-series analysis. IEEE Signal Process. Lett..

[6] Li Y., Li G., Yang Y., Liang X., Xu M. (2017). A fault diagnosis scheme for planetary gearboxes using adaptive multi-scale morphology filter and modified hierarchical permutation entropy. Mech. Syst. Signal Proc..

[7] Yang C., Jia M. (2021). Hierarchical multiscale permutation entropy-based feature extraction and fuzzy support tensor machine with pinball loss for bearing fault identification. Mech. Syst. Signal Proc..

[8] Li W., Shen X., Yaan L. (2019). A comparative study of multiscale sample entropy and hierarchical entropy and its application in feature extraction for ship-radiated noise. Entropy.

[9] Aboy M., Cuesta-Frau D., Austin D., Mico-Tormos P. Characterization of sample entropy in the context of biomedical signal analysis. Proceedings of the 2007 29th Annual International Conference of the IEEE Engineering in Medicine and Biology Society.

[10] Jiang Y., Mao D., Xu Y. (2011). A fast algorithm for computing sample entropy. Adv. Adapt. Data Anal..

[11] Mao D. (2008). Biological Time Series Classification via Reproducing Kernels and Sample Entropy. Ph.D. Thesis.

[12] Schreiber T., Grassberger P. (1991). A simple noise-reduction method for real data. Phys. Lett. A.

[13] Theiler J. (1987). Efficient algorithm for estimating the correlation dimension from a set of discrete points. Phys. Rev. A Gen. Phys..

[14] Manis G. (2008). Fast computation of approximate entropy. Comput. Meth. Prog. Bio..

[15] Manis G., Aktaruzzaman M., Sassi R. (2018). Low computational cost for sample entropy. Entropy.

[16] Wang Y.H., Chen I.Y., Chiueh H., Liang S.F. (2021). A Low-Cost Implementation of Sample Entropy in Wearable Embedded Systems: An Example of Online Analysis for Sleep EEG. IEEE Trans. Instrum. Meas..

[17] Liu W., Jiang Y., Xu Y. (2022). A Super Fast Algorithm for Estimating Sample Entropy. Entropy.

[18] Garivier A., Moulines E. On upper-confidence bound policies for switching bandit problems. Proceedings of the International Conference on Algorithmic Learning Theory.

[19] Anderson T. (2004). Towards a theory of online learning. Theory Pract. Online Learn..

[20] Silva L.E.V., Senra Filho A.C.S., Fazan V.P.S., Felipe J.C., Murta L.O. (2016). Two-dimensional sample entropy: Assessing image texture through irregularity. Biomed. Phys. Eng. Express.

[21] da Silva L.E.V., da Silva Senra Filho A.C., Fazan V.P.S., Felipe J.C., Murta L.O. Two-dimensional sample entropy analysis of rat sural nerve aging. Proceedings of the 2014 36th Annual International Conference of the IEEE Engineering in Medicine and Biology Society.

[22] Audibert J.-Y., Munos R., Szepesvári C. (2009). Exploration–exploitation tradeoff using variance estimates in multi-armed bandits. Theor. Comput. Sci..

[23] Zhou D., Li L., Gu Q. Neural contextual bandits with ucb-based exploration. Proceedings of the International Conference on Machine Learning.

[24] Gupta N., Granmo O.-C., Agrawala A. Thompson sampling for dynamic multi-armed bandits. Proceedings of the 2011 10th International Conference on Machine Learning and Applications and Workshops.

[25] Cheung W.C., Simchi-Levi D., Zhu R. Learning to optimize under non-stationarity. Proceedings of the 22nd International Conference on Artificial Intelligence and Statistics.

[26] Xu M., Qin T., Liu T.-Y. Estimation bias in multi-armed bandit algorithms for search advertising. Proceedings of the 26th International Conference on Neural Information Processing Systems.

[27] Sarwinda D., Paradisa R.H., Bustamam A., Anggia P. (2021). Deep learning in image classification using residual network (ResNet) variants for detection of colorectal cancer. Procedia Comput. Sci..

